# The effect of different concentrations of chlorine-containing disinfectants on high-frequency contact table in intensive care units: A quasi-experimental study

**DOI:** 10.1371/journal.pone.0281802

**Published:** 2023-02-15

**Authors:** Jing Li, Yanhua Zhang, Zhiyun Zhang, Haixia Zhang, Xiaojuan Wang, Fuchuan Wang, Hongyu Wang, Meiling Chen

**Affiliations:** 1 Obstetrics/Beijing Ditan Hospital Capital Medical University, Beijing, P.R. China; 2 Intensive care unit/Beijing Ditan Hospital, Capital Medical University, Beijing, P.R. China; 3 Nursing Department/Beijing Ditan Hospital, Capital Medical University, Beijing, P.R. China; 4 Medical Record Statistics Department/Beijing Ditan Hospital, Capital Medical University, Beijing, P.R. China; Universita degli Studi di Siena, ITALY

## Abstract

**Background:**

The hospital environment, particularly the intensive care unit (ICU), contributes to the transmission of several nosocomial pathogens, which can survive in this setting for a longer period of time and, in turn, contaminate the surfaces or the medical tools. Thus, appropriate disinfection of these areas and devices are crucial for controlling and preventing further infection. In this study, we examined the effect of different concentrations of chlorine-containing disinfectants (500mg/L, 1000mg/L, and 2000mg/L) on the ICU environment.

**Methods:**

This quasi-experimental study was based on a convenient sampling method. In this study, High-frequency objects were selected as subjects in ICU, with a total sample of 216.A hall including 6 beds was examined,selecting 4 high-frequency surfaces per bed unit:a bed gear, infusion system, bed end table, and monitor were disinfected with 500, 1000, and 2000 mg/L of chlorine (as Cl2), respectively.The surface dissection was performed at 21:00 o’clock daily, after which ATP fluorescence monitoring and bacterial count detection were performed.

**Results:**

There was no significant difference in ATP bioluminescence (F = 2.03, P > 0.05) and bacterial counting (χ2 = 2.03, P > 0.05) when using different concentrations of chlorine-containing disinfectant in the ICU. Yet, compared with high concentration (2000mg/L), a low concentration disinfectant reduced the hospital cost.

**Conclusion:**

By reducing the concentration of ICU high-frequency contact table disinfectants, it is possible to reduce the risk of long-term contamination with chlorine-containing disinfectants and reduce the cost of using ICU chlorine-containing disinfectants.

## Introduction

Nosocomial infections are very common among ICU patients [1]. The surfaces and equipment of the ICU, which are the major source of infections, may be contaminated by bacteria; thus, medical institutions are required to clean and disinfect the surface at least once a day [2, 3]. Maintaining the environment clean is the primary condition for controlling and preventing infections in the ICU [4, 5]. "Technical Specification for Disinfection of Medical Institutions" (WT/T 367–2012) guidelines [6] recommend using 400mg/L-700mg/L available chlorine-containing chlorine wipe with disinfectant. Yet, in some hospitals, a much higher concentration of chlorine-containing disinfectants is used to prevent serious infections. Previous studies have reported that high concentrations of chlorine-containing disinfectants (>2000 mg/L) can increase damage to the environment and the surface of objects and instruments.First, a chlorine residual as low as 0.91 mg/L as Cl2 may cause developmental toxicity in a polychaete P. dumerilii in receiving water body [7]. Second, the high chlorine residual in wastewater is required to be dechlorinated, which involves the cost of a dechlorinating agent [8]. Third, the high chlorine residual in wastewater may react with organics in wastewater to form harmful halogenated disinfection byproducts, which may cause developmental toxicity and growth inhibition on the aquatic creatures in receiving water body [9, 10]. In this study, we examined the effect of different concentrations of chlorine-containing disinfectants (500mg/L, 1000mg/L, and 2000mg/L) on the ICU environment.

## Methods

### Study design

This quasi-experimental study was based on a convenient sampling method. In this study, High-frequency objects were selected as subjects in ICU, with a total sample of 216.A hall including 6 beds was examined,selecting 4 high-frequency surfaces per bed unit: a bed gear, infusion system, bed end table, and monitor were disinfected with 500, 1000, and 2000 mg/L of chlorine (as Cl2), respectively. The frequency of wiping and disinfection of the high-frequency content in hospital is 3 times a day, namely 05:00o’clock,13:00o’clock and 21:00o’clock, to maintain the effective disinfection effect, and quality passed for each wipe disinfection.This study was conducted at 21:00o’clock based on 13:00 wipe disinfection.(as the initial level of microbial contamination)The surface dissection was performed at 21:00 o’clock daily, after which ATP fluorescence monitoring and bacterial count detection were performed.

Pathogen sampling was carried out in strict accordance with the Technical Specifications for Disinfection of Medical Institutions (WS/T367-2012) and the Hospital Disinfection Hygiene Standards (GB15982-2012).

### Randomization and blinding

According to Huslage’s list classification, and considering the actual situation of the hospital [11], the ICU was reasonably clothed. The identification location selection principle, in order to avoid the effects of the natural removal of patients, medical personnel, etc., is not directly identified on the list but on the contact location of the table that needs to be cleaned and disinfected. Double-blind survey. The investigation plan is limited to the hospital infection management department, and investigators know, the specific implementation department and cleaning staff in advance without notice.

### Quality control

The quality control included the following: (i) inspectors received special training; the position, shape, and size of the dots were unified; identification point removal criteria were reviewed; places that are hard to naturally remove were selected (to draw a square (the side length is about 2 cm)). (ii) Standardized disinfection and wiping requirements: fixed wiping personnel (wiping personnel are trained to identify correct cleaning and disinfection methods and determine cleaning intensity and frequency, and those who passed the assessment could join this study after training.), wearing disposable isolation gowns, disposable medical surgical masks, disposable caps, and disposable medical gloves (changing gloves every time a bed is wiped). Each bed was equipped with a special disinfection bucket, measure cup, and towel (towels are divided into 4 colors: green towel wipe infusion system, blue towel wipe monitor, yellow towel wipe bed table, and pink towel wipe bed file). Wiping order: (1) infusion system; (2) monitor, (3) bed end table, and (4) bed file. (iii) Standardize the configuration of chlorine-containing disinfectants: chlorine-containing disinfectants were prepared with water of ≤20°C within the validity period. Before wiping, the towel was immersed in chlorine for more than 10 minutes. After the test was completed, considering that the disinfection concentration of the chlorine-containing disinfectant 500mg/L and 1000mg/L cannot be detected in time after wiping, in order to ensure that the risk of nosocomial infection will not increase, then wipe with the chlorine-containing disinfectant of 2000mg/L.

### Outcomes

The primary endpoint indicators were: (i) biofluorescence detection was performed as previously described [12]. Briefly, the ATP bioluminescence consisting of a swab was placed in a tool that uses the firefly enzyme luciferase to catalyze the conversion of ATP in AMP. This reaction then emitted light (560nm fluorescence), which was detected by the bioluminometer. The testing product was obtained from Beijing Luvof Medical Technology Co., Ltd. (No. 345ATP, 345US). Higher ATP content indicates higher bacterial content (the ATP test results on the surface of the object of less than 50RLU/swab were considered qualified, while 50RLU/swab were considered not qualified). (ii) Bacterial culture [13]: sampling tube was mixed using a vortex mixer for 20s. Then, a 1mL sample was placed into an empty flat dish with a diameter of 90mm containing melted nutritional agar (45–48°C,16 -18mL). Plates were then cultured at 35° C in the incubator culture for 48h, after which the bacteria were counted. Samples were tested in the Colombian plate at 35 degrees CO_2_ incubator culture 18–24. Evaluation standards were performed in accordance with the People’s Republic of China "hospital disinfection hygiene standards" (GB15982-2012) requirements; Class II environmental objects surface bacterial ≤5cfu/cm^2^ reach the standard.

The secondary endpoint indicator was: cost/year of use of chlorine-containing disinfection raw liquid = the amount of chlorine-containing disinfection raw liquid × 0.014 Dollars/10ml×365.

### Statistical analysis

SPSS20.0 statistical software was used for data analysis. The measurement data is represented as $\left(\bar{x}\pm s\right)$. Different concentrations of chlorine disinfectant comparison and bacterial culture results were analyzed using Fisher’s precision test, while the biofluorescence test results were examined using a t-test. *p*>0.05< 0.05 was considered to be statistically significant.

## Results

### Biofluorescence detection results

**Fig 1:**
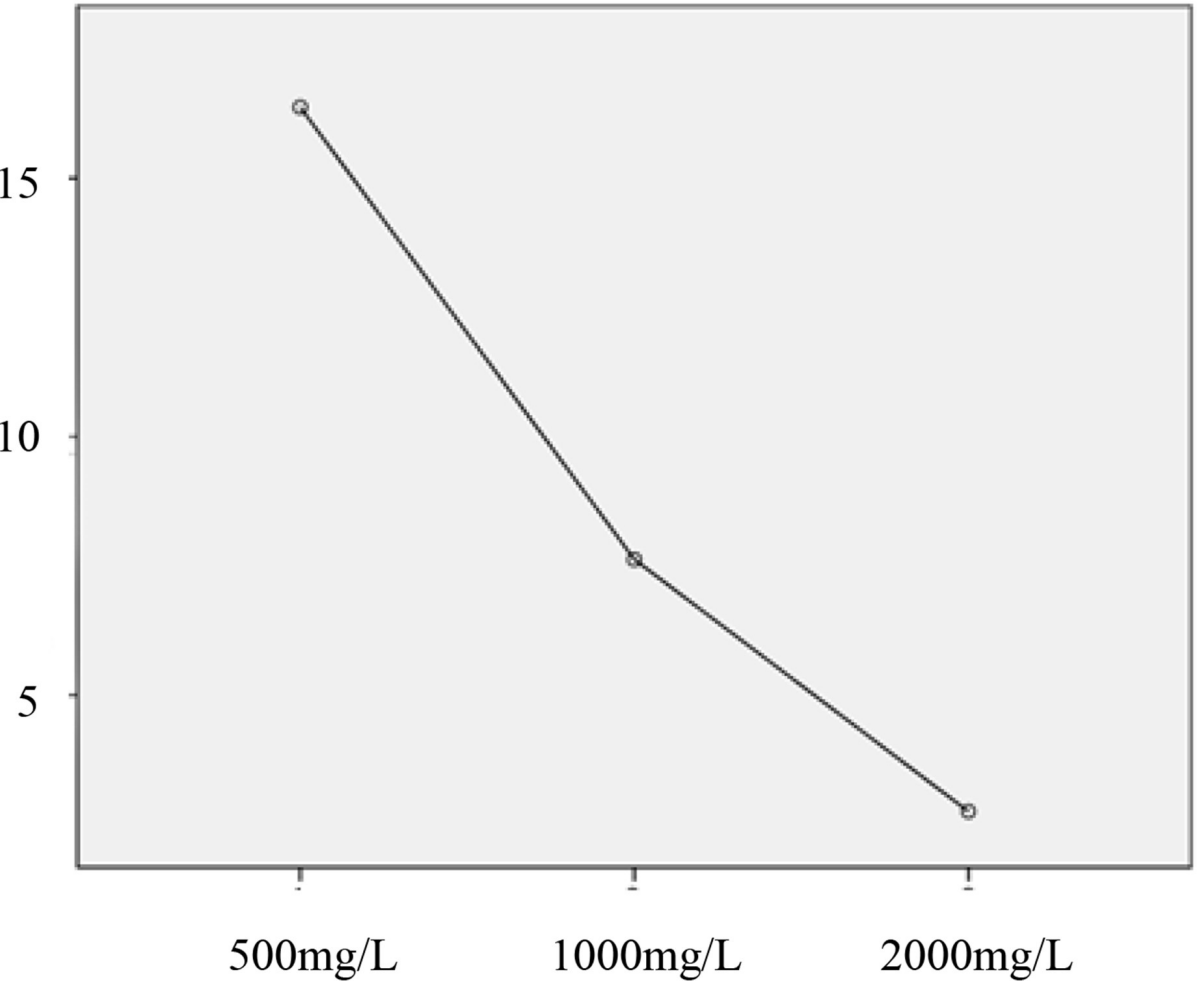
Test mean plot. The mean value diagram of bioluminescence assay for high-frequency objective indicators with different concentrations.

After wiping with 500mg/L chlorine-containing disinfectant, the ATP was 16.37±58.33RLU/swab. The unqualified areas were the bed gears and the end table. After wiping with 1000mg/L chlorine-containing disinfectant, the ATP was 7.62±27.26RLU/swab. The unqualified area was the bed and infusion system. After wiping with 2000mg/L chlorine-containing disinfectant, the ATP was 2.75±5.52RLU/swab. There was no statistical difference between the 3 groups (F = 1.64, *p*>0.05) (**Fig 1**).

### Bacterial culture test results

Among 216 sample objects, 213 (98.61%) reach the standard, 3 (1.39%) unreach (2 results using 500mg/L chlorine-containing disinfectant to wipe, 1 result using 2000mg/L chlorine-containing disinfectant to wipe); the unreach areas included bed stalls and bed end tables. There was no statistically significant difference in the disinfection effect of different concentrations of chlorine-containing disinfectant (χ^2^ = 2.03, *p*>0.05) (**Table 1**).

**Table 1:** Bacterial culture results from different concentrations of high-frequency substances.

Concentration (mg/L)	Reach the standard n(%)		Chi-square value	*p*
	Yes	No		
500	70(97.22)	2(2.78)	*2.03	0.36
1000	72(100)	0(0)		
2000	71(98.61)	1(1.39)		
total	213(98.61)	3(1.39)		

*Result of Fisher’s precision probability test.

### Comparison of the disinfection costs

Next, we compared the disinfection cost of three chlorine-containing disinfectants on high-frequency contact objects per bed per day in the ICU. Compared with high concentration (2000mg/L), a low-concentration disinfectant reduced the hospital cost (**Table 2**).

**Table 2:** Comparison of disinfection costs of 3 chlorine-containing disinfectants per bed per day.

Concentration (mg/L)	Disinfectant (Dollars/10mL)	Per bed per day Dosage (mL)	Per bed per day Total (Dollars)	20 beds per year Total (Dollars)
500	0.014	20	0.029	215.204
1000	0.014	40	0.058	430.408
2000	0.014	80	0.117	860.816

## Discussion

Healthcare-associated infection (HCAI) has become one of the more serious problems worldwide. In the United States, 648,000–1.7 million hospitalized patients are affected by HCAI each year [14]. In 2004, the National Nosocomial Infection Surveillance System (NNIS) reported an incidence of HCAI in the ICU of 25%-33% in the United States [15]. Otter *et al*. suggested that surface and medical devices have an important role in the transmission process of some HCAI pathogens [16]. Thus, effective cleaning and disinfection of the ICU environment, especially of the surface of high-frequency contact objects, is particularly important [17]. The "Management Regulations for Environmental Surface Cleaning and Disinfection in Medical Institutions" stipulates that the environmental surfaces of high-frequency contact in the ICU shall be regularly disinfected. However, at present, the disinfection concentration and frequency of ICU in most hospitals do not follow a standard protocol and different methods and concentrations of disinfectants are applied by different units and countries [18]. In this study, we explored the optimal disinfection concentration for high-frequency contact surfaces of the ICU, which may help formulate practical disinfection plans and provide a useful reference for hospital infection management.

We applied two detection methods: ATP biofluorescence and bacterial counting. There was no difference in the disinfection effect of the high-frequency surface of the ICU when using different concentrations of chlorine disinfectant. Chlorine-containing disinfectant is dissolved in water to produce hypochlorous acid, and its active ingredient is available chlorine [19]. At present, medical institutions use“84”disinfectant with hypochlorite as the main component, which has a quick effect but poor stability. The US Centers for Disease Control and Prevention recommends using 5% sodium hypochlorite on the surfaces of objects that patients frequently touch [20]. The higher the effective chlorine content, the stronger the disinfection effect. However, frequent use of high-concentration disinfectants for disinfection may result in disinfectant resistance [21, 22]. Moreover, chlorine-containing disinfectant has corrosive and bleaching effects and can irritate the skin [23]. In addition, its inhalation can have e serious effect on the respiratory tract [24]. Therefore, a disinfection program with a low-concentration chlorine-containing disinfectant was adopted by ICU. While ensuring the disinfection effect of the ICU’s high-frequency surface reduces the risk of long-term pollution to the staff’s health and the environment.

In this study, we also compared the cost-effectiveness of chlorine-containing disinfectants. Compared with high concentration (2000mg/L), a low concentration disinfectant reduced the cost by 711.942 Dollars per year. In the environment of medical reform, medical institutions need to improve relevant measures to reduce hospital costs, which is conducive to realizing the economic and social benefits of the hospital [25]. Further studies should investigate how the ICU can ensure the disinfection effect of high-frequency contact surfaces while minimizing consumption.

Limitations of the study:(i)Because this was a quasi-experimental study with a single-center, small sample, it was somewhat limited. In the future, larger sample data sets will be required to confirm and validate the experimental results. (ii)Due to the nature of the research, we did not conduct a detailed microbial study. We intend to investigate (i)selecting sensitive disinfectants based on pathogenic bacteria resistance characteristics, ensuring disinfectant concentration and action time, and killing pathogenic strains all at once in the following study. (ii)For comparative analysis, choose a variety of disinfectants as well as one fixed disinfectant.

In conclusion, reducing the concentration of disinfectant on the surface of ICU high-frequency contact objects does not affect the disinfection effect but helps reduce the cost of clinical use. In unqualified cases, they are found to be mostly concentrated on high-frequency contact surfaces, such as bed rails and bedside tables, and the unit of the patient bed is closely related to the spread of pathogenic microorganisms in the hospital [26]. The department formulates detailed, diversified, and targeted improvement measures based on the characteristics of unqualified cases, the number of users, and other factors. In our future study, we hope to develop a unified and perfect clean workflow and system, establish a standardized and refined evidence-based medicine-based surface management strategy and specification for high-frequency contact environmental objects, and realize the accurate management of infection control in the medical environment [27].
